# Risk Perception: Chemical Stimuli in Predator Detection and Feeding Behaviour of the Invasive Round Goby *Neogobius melanostomus*

**DOI:** 10.3390/biology13060406

**Published:** 2024-06-02

**Authors:** Natalia Z. Szydłowska, Pavel Franta, Marek Let, Vendula Mikšovská, Miloš Buřič, Bořek Drozd

**Affiliations:** South Bohemian Research Center of Aquaculture and Biodiversity of Hydrocenoses, Faculty of Fisheries and Protection of Waters, University of South Bohemia in České Budějovice, Zátiší 728/II, 389 25 Vodňany, Czech Republic; pfranta@frov.jcu.cz (P.F.); mlet@frov.jcu.cz (M.L.); vmiksovska@frov.jcu.cz (V.M.); buric@frov.jcu.cz (M.B.); drozd@frov.jcu.cz (B.D.)

**Keywords:** alarm cues, *shreckstoff*, food consumption, gut evacuation rate, predation efficiency, aquatic invasions, non-native species

## Abstract

**Simple Summary:**

The round goby is considered one of the most widespread Ponto-Caspian invasive species, which poses a threat to ecosystems and food web alterations. While it is known for direct predation, its interactions with native higher predators and its feeding behaviour upon their presence remain unclear. Therefore, we investigated how the exposure to chemical alarm cues emitted by conspecifics and the odour of a native predator, the European eel, affects the round goby’s feeding activity. Surprisingly, the gobies did not exhibit any pronounced threat sensitivity to both types of chemical stimuli, which was observed in unchanged food consumption probability and gut evacuation rates. The obtained outcomes suggest boldness, great efficiency in food processing, and a potential competitive advantage over native counterparts when colonising new ecosystems, irrespective of local higher predator presence.

**Abstract:**

The round goby *Neogobius melanostomus* is a notoriously invasive fish originating from the Ponto-Caspian region that in recent decades has successfully spread across the globe. One of its primary impacts is direct predation; in addition, when entering new ecosystems, the round goby is likely to become a food resource for many higher native predators. However, little is known either about the indirect effects of predators on the round goby as prey or its feeding behaviour and activity. The non-consumptive effect of the presence of higher native predators presumably plays an important role in mitigating the impact of non-native round gobies as mesopredators on benthic invertebrate communities, especially when both higher- and mesopredators occupy the same habitat. We tested the food consumption probability and gut evacuation rates in round gobies in response to chemical signals from a higher predator, the European eel *Anguilla anguilla*. Gobies were placed individually in experimental arenas equipped with shelters and exposed to water from a tank in which (a) the higher predator had actively preyed on a heterospecific prey, earthworms *Lumbricus* sp. (the heterospecific treatment; HS); (b) the higher predator had fed on round gobies (the conspecific treatment; CS); or (c) the water was provided as a control treatment (C). To ensure exposure to the chemical stimuli, this study incorporated the application of skin extracts containing damaged-released alarm cues from the CS treatment; distilled water was used for the remaining treatments. No significant differences were observed in either the food consumption probability or gut evacuation rate in the tested treatments. Despite the lack of reaction to the chemical stimuli, round gobies did exhibit high evacuation rates (*R* = 0.2323 ± 0.011 h^−1^; mean ± SE) in which complete gut clearance occurred within 16 h regardless of the applied treatment. This rapid food processing suggests high efficiency and great pressure on resources regardless of the presence or not of a higher predator. These findings hint at the boldness of round gobies, which did not exhibit any pronounced threat sensitivity. This would seem to suggest great efficiency in food processing and a potential competitive advantage over local native species when colonising new ecosystems, irrespective of the presence of native predators. Our study did not detect any non-consumptive effect attributable to the higher predator, given that the feeding activity of the invasive round goby was not altered.

## 1. Introduction

Alien invasive species continue to pose a significant threat to biodiversity [1]. Their rising impacts may be attributable in many cases to globalisation [2,3] and progressive global climate changes [4,5] and have serious ecological and economic consequences [6,7,8]. In recent decades, the Ponto-Caspian region has emerged as a notable source of fresh species introductions throughout Europe [9], which include a significant number of representatives from the Gobiidae (Actinopterygii) family [10]. At least five species have established populations in European inland freshwater ecosystems [11], of which the most widespread and well-known is the round goby *Neogobius melanostomus* (Pallas, 1814). This fish has successfully expanded its range worldwide [12] and now serves as a model species for studying invasions in freshwater ecosystems [13].

As an invasive species, the round goby exhibits numerous specific traits that have facilitated its successful colonisation of new freshwater and marine ecosystems on both sides of the Atlantic Ocean [14,15,16,17]. Key among the characteristics facilitating its establishment is its opportunistic feeding strategy [18,19] that guarantees effective food resource exploitation. However, since many introduced species are not only predators but also prey for native species when they enter new ecosystems, the round goby’s ability to manage risks posed by unfamiliar predators might also be contributing to its invasive success [20,21].

When entering new ecosystems, the round goby notably disrupts community dynamics and food webs [22], primarily through direct predation on benthic invertebrate communities [23,24], as well as on fish fry and eggs [25,26]. Hence, depending on the round goby’s diet shifts during ontogeny [27], as well as seasonal and/or environmental conditions [28,29,30], different macroinvertebrate assemblages will experience intense predatory pressure leading to significant declines [31,32,33]. Additionally, intensified foraging on particular taxa and competition for habitat and food resources will further contribute to the depletion of native fish abundance and richness [34,35,36], thereby leading potentially to their replacement [37]. Moreover, besides its commonly attributed role as a predator, the round goby has become an essential dietary item for numerous native higher-predator fish species [38,39,40] and in some cases is even the main food item due to its long-term presence in the ecosystem [41].

These predator–prey interactions are crucial drivers of trophic dynamics and structural and functional changes within ecosystems [42]. Recent studies have shown that predators can exert threats and affect prey populations not solely via direct consumption but also indirectly [43,44]. Non-consumptive effects are observed when mesopredators face the trade-off between predation risk and other activities such as, for instance, resource acquisition [45]. Compared to the direct foraging effect, non-consumptive effects appear to be equally or even more significant [46], since they can affect the entire prey population by inducing adaptations of defensive strategies [47]. This can lead to modifications in fish morphology, physiology, and behaviour where, depending on the length of exposure to the risk (acute or chronic), the response of fish will differ [48].

Following that, the early detection of a local predation risk presence represents a keystone ability for prey survival [49,50]. Foragers gather information by perceiving and relying on a variety of stimuli [44,51], with chemical communication believed to be fundamental for risk assessment and prey avoidance [52,53,54,55] irrespective of environmental conditions [56]. These stimuli include stress-inducing cues resulting from prey–predator interactions [57]. Both predator and prey specimens can actively release these cues: predator-borne cues include kairomones and digestion-released (dietary) cues recognisable by conspecifics of the consumed prey [58,59], while prey-borne cues derived from epidermic injuries or active foraging serve as pre-attack alarm alerts that warn conspecifics of the threat of a potential attack [53,60,61,62]. As a result, all signals may lead to both behavioural [63,64,65] and physiological [59,66,67] anti-predatory responses, which are often specific to a given species [60,67]. Different fish species appear to respond differently to identical chemical pictures [68] or to the type of chemical signal to which they are exposed [69]. All of the abovementioned comprise adaptations that significantly contribute to increasing the prey survival rate [70].

Although significant advances have been made in studying predation on the round goby [71], our understanding of the indirect effects of the presence of higher predators on mesopredators (i.e., the round goby) remains limited. Therefore, in this study, we aimed to investigate whether or not round gobies respond to chemical stimuli and whether or not the presence of the local higher predator, the European eel *Anguilla anguilla* (Linnaeus, 1758), affects gobies’ feeding and digestion efficiency. We assessed this by counting the number of prey items consumed and by analysing evacuation rates (*R*), i.e., the mass of food expelled from the digestive tract per unit of time [72]. This evacuation rate parameter is commonly used in direct in situ methods to quantify food consumption, e.g., [73,74,75], since it regulates feeding rates [76]. 

Foragers have to confront trade-offs between predator avoidance and other fitness-related activities [61,77]. This can manifest itself in altered feeding behaviour [78] including shifts in feeding-hiding ratios [63,79], a reduction in food intake in fish exposed to the chemical cues provided by higher predators [80,81,82,83,84], or even complete feeding latency [65,69,85]. Thus, we expected to observe similar reactions in round gobies. In addition to recognising predator odours, we predicted that the round goby would respond sharply to alarm cues derived from conspecific individuals. Therefore, we hypothesised that exposure to chemical cues, i.e., predator odour and conspecific alarm cues, would significantly contribute to a specific anti-predatory response. This response was expected to manifest itself in the form of decreased foraging activity and slower gut evacuation rates due potentially to lower consumption rates [86].

## 2. Materials and Methods

### 2.1. Collection and Maintenance of Study Organisms

*N. melanostomus* (a non-native mesopredator) individuals in a size range 55–75 mm were collected in the river Elbe (Dolní Žleb, North Bohemia, Czech Republic GPS: 50.50335 N, 14.13019 E) by electrofishing (a backpack pulsed-DC electrofishing unit FEG 1500, EFKO, Leutkirch, Germany) (~0.5 m water depth; 0.02–0.49 m s^−1^ water velocity). Fish capture was conducted in a recently established population first documented by Buřič et al. [87]. Fish were transported to the experimental facilities of the Institute of Aquaculture and Protection of Waters (FFPW USB) in České Budějovice where they were acclimatised in a recirculating aquaculture system (RAS) with a total volume of 1600 L for at least one month before the experiments were conducted. Selected physical–chemical parameters (T, O_2_, pH) were monitored using daily measurements conducted with an HQ40d digital multimeter (Hach Lange GmbH, Düsseldorf, Germany).

*A. anguilla* (a native higher predator) were captured at another locality on the river Elbe (Lovosice, North Bohemia, Czech Republic; 50.5165686N, 14.0595781E), where the co-occurrence of these two species—as well as eel predation on round gobies—has been confirmed (obs. pers.). Given the shared habitat [88] and the similar temperature conditions required for growth and consumption [89,90,91], the European eel was chosen as the higher predator for our experiment. Eels sized 540–840 mm (known to feed on small-size fish species including round gobies; [92,93]) were transported to the facility and stocked in the two separate recirculating systems (RAS; 2 × 1600 L) and were subsequently used in the experiment. For at least one month before the experiment began, the eels were fed on round gobies (first group, representing the conspecific treatment; CS) or on common earthworms *Lumbricus terrestris* (Linnaeus, 1758) (second group, representing the heterospecific treatment; HS) to acclimatise them to the food to be used during the experiment and to ensure adequate prey intake. All the abovementioned physical–chemical parameters were controlled daily.

The experimental food provided for the round gobies included *Chironomus* sp. larvae (frequently observed in their diet, [94,95]) purchased from a pet store and *Gammarus fossarum* (Koch in Panzer, 1835), selected as an alternative dietary source that is easily distinguishable from the tested food even after digestion. The *Gammarus fossarum* individuals (successfully preyed upon by round goby, [96]) were collected from Dobrovodský potok (Třebotovice, South Bohemia, Czech Republic; 48.9507 N, 14.5676 E), frozen on-site using dry ice, and subsequently stored at −80 °C until the beginning of the experiment. 

### 2.2. Preparation of the Alarm Cues

The preparation of the alarm cues used in this study followed the methods described by Wisenden et al. [61]. Since it was unfeasible to guarantee a predation event at a specific time, to ensure the efficacy of the experiment and to strengthen the effects of the alarm cues, we created an extract from round goby skin containing alarm pheromones released from epidermal cells while fish are attacked/injured [97]. This allowed us to simulate the release of alarm signals without relying solely on spontaneous attacks by higher predators. The 30 donor round gobies in a size range of 71–90 mm (81.3 mm ± 5.5 mm, TL ± SD) were sacrificed by a blow to the head to prevent any potential interference in the release of chemical signals that could occur if anaesthetics were used. A skin fillet was carefully removed from both sides of each fish to obtain approximately 130.5 cm^2^ of skin devoid of muscle tissue. All skin samples were promptly placed in 200 mL of distilled water, kept chilled on ice to prevent any decomposition until further processing, and homogenised. Subsequently, the homogenised samples were filtered through a glass-wool filter twice to remove any remaining particles (e.g., scales) and then diluted with distilled water to form a solution with a final concentration of 0.1 cm^2^ of skin.ml^−1^ [61]. Subsequently, syringes with a volume of 5 mL were filled with the prepared extract and stored in the freezer at −29 °C before use in experiments.

### 2.3. Experimental Design

Based on the modified methodology described by Richter et al. [98], as well as our own previous experiences, we set up a series of five experimental trials (see below) to examine the gastric evacuation rate and food consumption probability under three different treatments. These treatments involved exposing tested fish to different conditions: (a) conspecific alarm cues and kairomones released by a higher predator fed on round gobies (conspecific treatment; CS); (b) chemical cues released by a higher predator fed on earthworms (heterospecific treatment; HS); and (c) clear water devoid of any chemical stimulants (control treatment; C).

**Figure 1:**
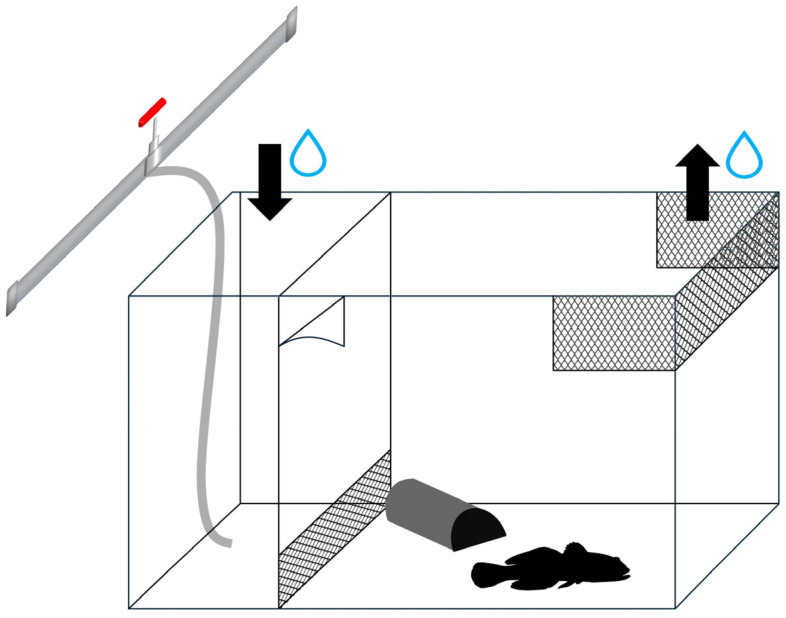
Experimental arena. Black arrows mark water inflow and outflow, respectively.

Each treatment was tested in a parallel trial (five repetitions) within a separate recirculation system comprising four vertically arranged tanks, each with a capacity of 400 L (RAS; 4 × 400 L). The uppermost and lowermost tanks were fitted with biological and mechanical filters in each system, respectively. Depending on the treatment, the upper tank was stocked either with seven European eels fed on round gobies (whose numbers were controlled by the end of each experimental trial; CS treatment), seven European eels fed on earthworms (HS treatment), or no European eels (chemical cue-free water; C treatment). The water containing specific chemical cues flowed into the second lower tank, where 11 experimental arenas per treatment were placed, i.e., 33 arenas for each experimental trial were used at the same time. Each experimental arena consisted of an aquarium (total volume of 6.5 L) divided into two parts by a glass wall with a lower steel mesh to maintain the water flow (Figure 1). The tested fish was placed in the larger section (23.0 × 19.5 × 20 cm) equipped with a half-cut pipe serving as a shelter and an upper outlet for water flow. The smaller section (10 × 23 × 20 cm) had an inlet for flowing water (Figure 1). Each aquarium was covered with a matte translucent film to minimise stress and disturbance and to prevent visual interactions between gobies.

Before each trial, a 36-h fasting period was imposed on the fish to ensure complete gut clearance and preclude any potential influence of the remains of the acclimatisation food on the experiment outcomes. To prevent possible cannibalism provoked by starvation and to ensure that a particular individual consumed a given amount of food, all fish were placed separately in experimental arenas. The light regime was set to 12 h light–12 h dark, including dusk and dawn simulations. The temperature was maintained at a constant rate throughout the experiment at 21.48 ± 0.026 °C; mean ± SE. Automatic dataloggers Minikin T (EMS, Brno, Czech Republic) monitored the temperature every two hours.

**Figure 2:**
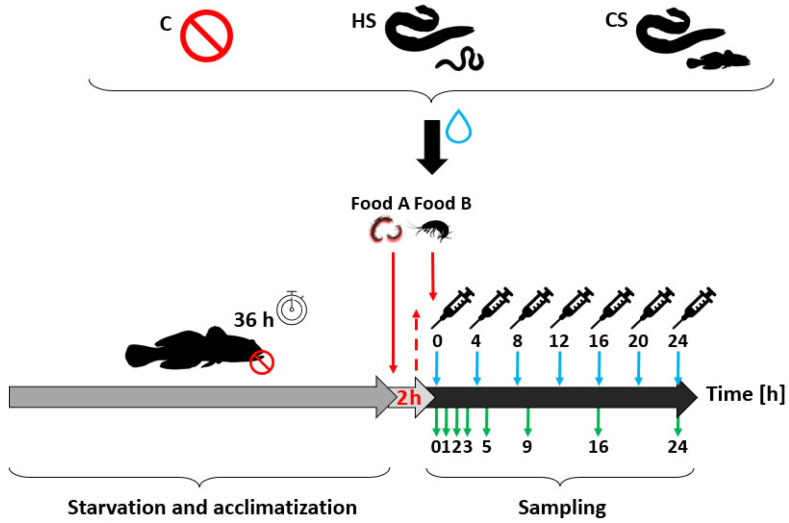
Experimental setup. C, control treatment (supplied by clear water without any chemical cues derived from predator or prey), HS, heterospecific treatment (supplied by water from a tank with predator fed by common earthworms), CS, conspecific treatment (supplied by water from a tank with predator fed by round gobies); solid red lines depict the application of food A (*Chironomus* sp. larvae) and food B (*Gammarus fossarum*); the dashed line represents the removal of the remains of food A; blue arrows represent the hours at which the chemical alarm cues (skin extract from the round goby in CS treatment) or distilled water (in C and HS treatments), depending on the treatment, were applied; green arrows denote the time intervals after feeding with food B at which the tested fish were sampled.

To obtain the conspecific cues, 15 round gobies were introduced into the tanks where the eels had been feeding before the start of each trial. After each trial, tanks were replenished with gobies to ensure an equal number at the start of each trial. Following a 36-h starvation period, the round gobies were fed with the tested food (Food A) consisting of 12 *Chironomus* sp. larvae with an average whole portion weight of 0.074 g ± 0.023 g, (W ± SD) (see Figure 2), which were used to observe the evacuation rate of the fish. After two hours, the tested food remains were removed from each aquarium, which marked the start of the main experimental phase and sampling. Fish that did not consume any test food within this period were excluded from the remaining part of the experiment. At this time, easily distinguishable food, marked as Food B, was given to the fish to observe the evacuation rate while continuous feeding was applied [99]. Simultaneously, the external stimulant, i.e., the predator odour from the upper tank and prepared alarm cues or distilled water, were added to the treatments to observe the consumption probability of food B and the effects of the chemical cues on the evacuation rate of food A. Thus, four individuals of *Gammarus fossarum* (food B) with an average whole portion weight of 0.069 g ± 0.062 g (W ± SD) were provided to all experimental round gobies. Tubes were placed in the aquariums to allow clean water or water containing chemicals from the eel tanks to flow in throughout the experiment. Simultaneously, the first dose (5 mL) of alarm cues (for the conspecific treatment) or distilled water (for the heterospecific and control treatments) was released. To ensure continuous exposure to the chemical signals, which have been shown to persist in aquatic environments for up to six hours [100], the prepared solutions were released repeatedly every four hours (Figure 2). The experimental arenas were checked simultaneously to ensure that constant food availability was being maintained.

Fish samples were taken at eight time intervals: 0, 1, 2, 3, 5, 9, 16, and 24 h after feeding with food B (Figure 2). In total, we tested 144 fish specimens across five trials of the experiment, with six replications at each time point. When sampling each round goby, the number of prey individuals remaining (food B) in the experimental arena was counted to estimate the total amount of food consumed by the round goby individual over a given time. The collected fish were killed using the anaesthetic MS 222 and kept in a freezer at −80 °C until further analysis, conducted within two weeks of completing the experiment.

### 2.4. Sample Processing

Before determining the gut contents of the round goby individuals, several measurements were taken, including total length (TL; mm), standard length (SL; mm), body weight with a weighing accuracy of 0.01 g (W; g) (Kern and Sohn GmbH, Balingen, Germany), and sex determination [15]. Subsequently, each fish was dissected, with the gut extraction following the methodology outlined by Manko [101]. The extracted gut/intestine and its contents were weighed to an accuracy of 0.1 mg (EX224M analytical balance, OHaus Corporation, Parsippany, NJ, USA). The length of the gut was measured, and the position of each food type (foods A and B) was observed. Next, the gut contents (food A and B separately) were placed on glass microfibre discs (type 691, 516–0862; VWR International, Radnor, PA, USA) that had been preheated in a laboratory chamber furnace (LAC-Ht40A1; LAC s.r.o., Židlochovice, Czech Republic) to completely decompose all organic carbon molecules (30 min at 500 °C). All the microfibre discs were weighed (Mettler-Toledo Excellence Plus XP6 microbalance; Greifensee, Switzerland) before the gut content was placed upon them. The empty intestine was also weighed and subsequently placed on pre-numbered and weighed aluminium foil. The body of the de-gutted fish (portioned to ease the drying process) was weighed (M3P, Sartorius, Goettingen, Germany) and placed on aluminium foil alongside the empty gut. Finally, samples of the gut contents (food A and B), the fish carcasses, and the empty dissected gut were dried overnight at 105 °C and stored in a desiccator to determine the dry weight (DW) using microbalances.

### 2.5. Data Analysis

All data analyses and graphical procedures were performed using R software (R Development Core Team, v. 4.1.1, Vienna, Austria).

#### 2.5.1. Assumptions of Observation Independence

We tested the assumption that all 24 design-based groups represented by round goby individuals sampled at eight individual sampling time points for each treatment did not differ either in standard length (SL) or wet weight (WW). Generalised linear models (GLMs) were employed with Gamma distribution and a ‘log’ link function. In addition, the differences between these design-based groups in the counts of the quantity of food A consumed were performed using a GLM with Poisson distribution and the ‘log’ link function. Due to the underdispersion detected, a quasi-likelihood estimation method was applied.

#### 2.5.2. Gut Evacuation Rate

The evacuation rate was estimated with statistical modelling of the weight of the prey remaining in the gut over time, whereby the relative gut content (*GI*) was calculated as a ratio of the dry weight (*DW*) of the tested food (food A) at the time of sampling vs. fish dry body weight. Subsequently, based on (*GI*), we computed the evacuation rate (*R*). This parameter can be described using linear and/or exponential functions [72,74]. Hence, given the uncertainty presented within the literature, we fitted our data to both linear Equation (1) and exponential Equation (2) models, represented by the following:

(1) $$GI_{t}=GI_{0}-Rt$$

(2) $$GI_{t}=GI_{0}e^{-Rt}$$

where *GI_t_* is relative gut content at time *t*, *GI*_0_ is the initial intestine content at postprandial time *t*_0_ (h), and *R* is the gastric evacuation rate [74,102].

We fitted the equations using GLMs with Gaussian distributions; ‘identity’ and ‘log’ link functions were applied. We tested the relationship between the relative gut content *GI* used as a response variable and the explanatory variables: (I) ‘time’ (continuous numerical variable) and (II) ‘treatment’ (three-level factor). All zero values for the relative gut content were replaced by 0.0014 mg to represent half of the overall minimum gut content mass. The use of a Gaussian distribution was appropriate given that the histogram of model residuals was found to be close to the Gaussian; the assumption of homoscedasticity was also fulfilled. The quality of the model fits was expressed using the McFadden coefficient of determination (*R*^2^). Regression coefficients for each treatment yielded by the model with the higher *R*^2^ were taken as the individual evacuation rates.

#### 2.5.3. Consumption Probability

To test differences between treatments at all time points in the consumption probability of offered prey (food B), we employed a GLM with a binomial distribution with the logistic link function. The proportions of eaten and offered prey were used as a response, and observations were weighted by counts of offered prey. Due to the detected overdispersion, a quasi-likelihood estimation method was applied.

## 3. Results

A total of 144 round goby individuals (SL = 55.2 ± 2.51 mm; wet weight WW = 3.79 ± 0.56 g; mean ± SD) were tested during five experimental trials. Depending on the treatment, the round gobies were observed to eat 0.023 (C), 0.033 (CS), and 0.036 (HS) g of provided food B, which corresponds to 2.31% (C), 2.12% (CS), and 1.94% (HS) of the wet fish body weight.

### 3.1. Assumptions of Observation Independence

All 24 design-based groups were completely standardised in terms of round goby SL (*F*_23,120_ = 0.93, *p* = 0.56), WW (*F*_23,120_ = 0.88, *p* = 0.63), and amount of food A consumed (*F*_23,120_ = 1.30, *p* = 0.18).

### 3.2. Gut Evacuation Rate

**Figure 3:**
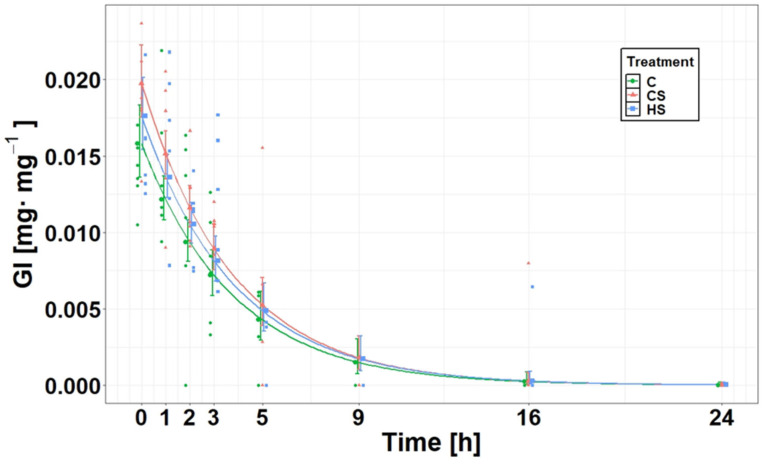
Relative gut content *GI* (computed as DW food A/DW fish) at the time after food application in the round goby (*Neogobius melanostomus*) under three experimental treatments: C, control treatment (no chemical cues); CS, conspecific treatment (conspecific alarm cues and kairomones released by a higher predator fed on round gobies); HS, heterospecific treatment (chemical cues released by a higher predator fed on earthworms).

**Figure 4:**
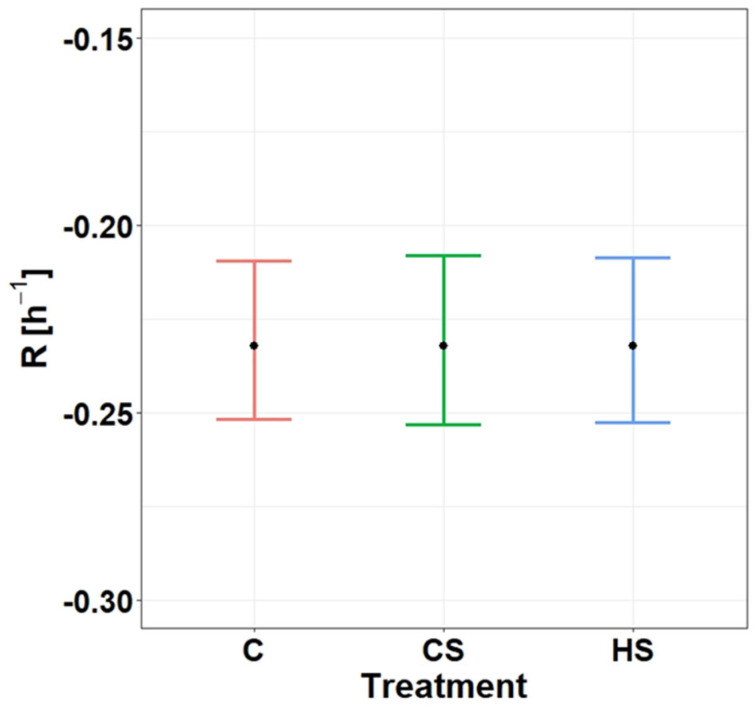
Overlap between the 95% confidence intervals of the gastric evacuation rates (R; mean ± S.D., n = 144) of the tested round gobies under three experimental treatments: C, control treatment (no chemical cues); CS, conspecific treatment (conspecific alarm cues and kairomones released by a higher predator fed on round goby individuals); HS, heterospecific treatment (chemical cues released by a higher predator fed on earthworms).

The fit of the exponential GLM Equation (2) was better than the fit of the linear GLM Equation (1) when comparing their *R^2^* values (exponential model: *R^2^* = 0.77; linear model: *R^2^* = 0.49). Both linear and exponential GLMs revealed that the interaction between ‘treatment × time’ (Model 1; Table 1) obtained by modelling the effects on *GI* was not significant (*p* = 0.61 and *p* = 0.96, respectively); thus, the evacuation rate was probably not influenced by the ‘treatment’. A further exponential GLM with additive effects (Model 2; Table 1) revealed a significant effect for both ‘treatment’ (*F*_2,140_ = 4.69, *p* = 0.01) and ‘time’ (*F*_1,140_ = 453.90, *p* < 0.001) on *GI*. *GI* in CS was 23.7% higher than in C and 9.8% lower in C than in HS (Figure 3). Nevertheless, complete gut clearance in all treatments took 16 h (Figure 3). According to the 95% confidence interval overlap, the evacuation rate was the same (*R* = 0.2323 ± 0.011 g·g^−1^ fish·h^−1^; mean ± SE given as an absolute value) in all treatments (Figure 4), which corresponds to the fitted decrease in *GI* per hour (20.7%). Since the linear GLM with additive effects did not reveal any significant effect of the ‘treatment’ (*F*_2,140_ = 1.43, *p =* 0.24) and considering the low effect size of the ‘treatment’ in exponential GLM Model 2 (Table 1), it is doubtful that the ‘treatment’ had any impact on the *GI*.

### 3.3. Consumption Probability

There were no significant effects of the interaction ‘treatment × time’ (*F*_12,105_ = 0.59, *p* = 0.85) (Table 2), as well as ‘treatment’ (*F*_2,117_ = 1.86, *p* = 0.16) or ‘time’ (*F*_6,117_ = 1.20, *p* = 0.31) (Table 2), indicating that the presence of the chemical stimulants did not significantly influence the probability of food B being consumed. 

## 4. Discussion

Our study revealed no discernible response in the invasive round goby to exposure to either type of chemical stimuli, and the feeding behaviour of the experimental groups subjected to different treatments was unaffected. The results of our experiment did not confirm the expected differences, including a decrease in the tested parameters due either to the presence of the predator’s odour or the chemical alarm signals received from injured conspecifics.

Despite the slightly lower amount of food B eaten in the HS treatment compared to the CS treatment (and especially C), all tested round gobies consistently processed and evacuated prey at the same rates per unit of time in all treatments, which is a surprising finding that contradicts our expectations. Considering that food processing (here represented by the evacuation rate) can be influenced by a multitude of factors including food properties [103,104] and predator and prey biometric characteristics [72,105,106,107], as well as by environmental conditions [108,109,110,111], we anticipated some variation between treatments. For instance, the presence of predators and their direct chemical recognition can lead to physiological activity changes [58,112] including alterations in hormone levels such as cortisol [113,114], as occurs in the case of conspecific disturbance or alarm cues [62]. In the context of our study, these physiological responses could be attributable to food intake and gut evacuation rates that are modified as stress-induced behaviour. However, although they did not differ, the treatment and time did seem to have a significant impact on the relative gut content. Nevertheless, considering the (i) small size of the statistical effect of the treatment, (ii) the lack of differences in the counts of consumed prey and fish biometrics between treatments, and (iii) the observation that all tested fish experienced complete gut clearance within 16 h regardless of the applied cues, we consider that the biological effect of treatment on the *GI* was negligible.

As for the evacuation rate, no significant differences were observed in the counts of the consumed prey (food B) (as given by the tested consumption probability). This also contradicts our expectations, as previous studies have demonstrated suppressed feeding activity including decreases in foraging and consumption rates under predator-induced stress conditions [62,87,115,116], as well as feeding latency [86] or even a complete cessation of food intake, as reported in the studies conducted by Giaquinto and Hoffmann [117]. However, since the complete reduction of food intake observed in the pintado catfish *Pseudoplatystoma corruscans* (Spix and Agassiz, 1829) was a result of the exposure to chemosensory information from non-injured conspecifics [117], we should question which alarm signals—i.e., disturbance or *shreckstoff*—are most important in overall conspecific communication. On the other hand, Fraser and Huntingford [118] proposed that foragers may exhibit various responses to danger, with ‘risk-reckless’ behaviour being one such mechanism whereby individuals ignore exposed hazards while maintaining maximum feeding levels regardless of food abundance. Our study sheds light on the behaviour of round gobies within this framework and suggests that it is an adaptation aiding its invasiveness success [119]. This behaviour matches previous research on round goby boldness [120,121,122] and how this trait influences its foraging ability [123,124]. It also facilitates the effective exploitation of available food resources with a visible superiority of bolder individuals over shy ones when it comes to resource usage [125] when invading new areas. While bold behaviour is often observed in specimens at the invasion front and is more characteristic of new populations rather than established ones [126], it is notable that bold fish also exhibit a smaller size-at-age compared to shy fish [127], which agrees with the overall size distribution of our tested fish population. Nevertheless, the inability to effectively balance predator avoidance behaviour with other fitness-related activities such as foraging may compromise fish survival and overall fitness, particularly in established populations [61,77,128]. Therefore, it is crucial to undertake further studies examining additional behavioural responses, shelter usage patterns, and potential shifts in round goby activity when encountering predators.

Given the existing evidence—i.e., increased respiratory responses [129]—that round gobies are responsive to chemical stimuli (specifically, injury-released alarm signals from conspecifics), we should examine whether or not the properties of the chemical cues and the intensity of the applied stimulants in our study were sufficient to elicit a response from the tested organisms. Our stimulus preparation followed the methodology outlined by Wisenden et al. [61] that uses the whole skin subjected to mechanical processing and extraction. The issue is whether another method [130] involving skin incisions washed with water could be more effective, as found in responses primarily in minnows. Many previous studies have shown that the magnitude of reactions in fish increases with the concentration of cues—e.g., in goldfish *Carassius auratus* (Linnaeus, 1758) exposed to dietary cues from a predator [131]—where variations in concentrations may indicate different degrees of threat [132]. Moreover, it seems that the frequency of the application of the alarm substance might also influence outcomes if we compare our results to those from studies of the behavioural response of the monkey goby *Neogobius fluviatilis* (Pallas, 1814) [133]. Continuous dosing with damage-released alarm cues (skin extract) in these studies may have given more potent effects than in our experiment in which the extract was only applied every four hours. However, we believe that our application time interval was sufficient since Hazlett [100] has reported that alarm cues fade from water within six hours; other studies have shown that the biological activities of injury-released chemical alarm cues in minnows and amphipods last for 3–6 h at 18 °C [134]. Even so, the round goby could be a less sensitive species characterised by a higher threshold regarding concentrations of alarm cues and/or the frequency of stimuli required to activate an expected response. Hence, other characteristics such as the persistence of the stimulus should be taken into account since multiple environmental conditions may influence particle breakdown and the degradation of alarm cues (solar radiation, temperature, pH, or oxygen levels) [130]. However, these factors may not be applicable to our study since we conducted them under laboratory conditions in which all parameters were maintained constant throughout the experiment. Since Chivers et al. [130] observed a decrease in the strength of the reaction intensity after 30 min, it seems that the initial application of the stimulants is key and each further application could acclimatise fish further to the chemical signal.

The sensitivity of fish in perceiving the threat signal is thus probably key. Responses to olfactory cues require the ability to perceive the specific intensity (concentration) of the odorants that compose the stimuli [135]. The sensitivity of olfactory receptors to specific chemicals varies between species, with observed specific threshold concentrations for cues [68] and fish at individual levels [136]. Specific variation is closely related to the anatomy and morphology of the olfactory organ [137], and a histochemical study has confirmed the widespread presence of olfactory sensory neurons in the peripheral olfactory organ, which underlines the crucial role of chemosensory signalling in round goby survival [138]. Thus, we anticipated that our tested fish would respond to the odour of predators as well as to alarm cues. Nonetheless, the whole Gobiidae family (Actinopterygii), which is known for the diversified production of alarm substances [139], exhibits variations in responses to chemical alarm communications despite the presence of epidermal club cells associated with such responses [140,141], a rule that our research seems to confirm. Thus, we believe that along with the perception threshold, another possible explanation of our observations may relate to the fact that chemical cue recognition in round gobies requires more experience and learning, as has been observed in many other fish [81,142]. Utne-Palm [143] claims that the recognition of odour in two-spotted gobies *Gobiusculus flavescens* (Fabricius, 1779) requires experience and that predator identities can be rapidly learnt by associating the smell of a predator with those of a prey chemical cue [144].

Regardless of the production of alarm substances, we did still expect to observe a reaction to the predator odour in the heterospecific treatment since the used prey was not in any way phylogenetically related to or belonged to the same prey guild [70,145,146,147,148,149,150,151,152,153,154,155,156,157,158]. However, unlike many invertebrates and amphibians, most fish are innately unable to recognise their predators’ kairomones, the exception being some groups such as salmonids [49,149]. Kairomones become associated with risk through direct exposure to alarm cues during a predatory attack or through the detection of dietary cues through passive [150], or even active [151], sampling behaviour during predator inspection. The ability of fish to respond to predators may not necessarily result from their ability to detect predator odours directly but rather from their ability to detect chemical alarm signals produced by conspecifics or heterospecifics in their diets. This has been confirmed in research on the pearl cichlid *Geophagus brasiliensis* (Quoy and Gaimard, 1824) by Arvigo et al. [69], which showed that predator odours did not induce any antipredator response. Similarly, the frillfin goby *Bathygobius soporator* (Valenciennes, 1837) is known to be indifferent to predator odours, although conspecific cues do affect its ventilation rate activity [66]. When exposed to predator odours and visual cues, the two-spotted goby likewise does not exhibit any innate anti-predator response [143].

On the other hand, research has demonstrated that even a single exposure to predator cues can enhance the learning process, thereby enabling fish to retain recognition of predators for extended periods and providing survival benefits. For instance, brook trout *Salvelinus fontinalis* (Mitchill, 1814) trained to recognise predators such as the chain pickerel *Esox niger* (Lesueur, 1818) have higher survival rates than untrained individuals [70]. Furthermore, learning could also play a critical role in acquiring experience with particular fish species and is required if the expected responses to predator cues are to be generated. However, given that the round goby and the European eel co-exist in the round goby source locality, we anticipate that a certain experience level would already have been established.

Our findings indicate that round gobies did not show any response to chemical stimuli from predatory odours combined with alarm substances, particularly those from conspecifics. This contradicts all expectations, as some fish species react to alarm substances produced by other species [152], with the most vital reactions typically provided by conspecific alarm substances [153]. However, little is known about fish anti-predator responses when predator and alarm cues are combined [69]. To date, anti-predator responses to the stimulants tested separately have been extensively studied in numerous fish species [60,65,154,155] showing that different types of chemical stimulants often induce different and specific responses [69]. Given this consideration, our approach using both chemical stimulants together may represent a limitation, so examining each stimulant separately could potentially provide a more complete understanding of the round goby’s chemical perceptions since one of the chemical cues might mitigate the impact of the other (although in this case, we believe it to be unlikely). However, it is essential to acknowledge that under natural conditions, chemical signals consist of a complex mixture of compounds conveying specific risk information [136,140]. Signals transmitted by predators combined with cues released during the digestion process can provide further detailed knowledge regarding the abundance, location, and recent feeding habits of predators in their environment [56]. Therefore, our method produced robust results, which may agree well with field-related conditions.

Most importantly, although our study did not reveal any changes in the food processing consumed between the tested groups, it is essential to emphasise that round gobies maintained consistently high evacuation rates regardless of the presence of higher predators. Combined with a previous study [99], these findings confirm the great efficiency of round gobies in processing and digesting food when continuously fed, particularly on chironomids. Given that evacuation rates closely regulate feeding rates [76], the rapid evacuation of chironomid larvae suggests potentially high predatory pressure on these macrozoobenthos taxa, present in the round goby diet [31,95,96], which could lead to modifications in benthic community composition and reduced resource availability for other consumers [23]. This trait, allied with the boldness of the round goby—i.e., obviously low sensitivity to predation danger—might suggest a potential competitive advantage over other local native species when colonising new ecosystems. For instance, compared to the ruffe *Gymnocephalus cernua* (Linnaeus, 1758), whose population decline has been linked to the appearance of this goby [36,156] as well as to a temporal collapse in ruffe populations [35,157], the round goby evacuates chironomid larvae faster under similar thermal conditions as those used in our studies. Moreover, the high predator-threat sensitivity in ruffes [158] suggests that the round goby could outcompete other species for food resources when introduced into a new environment, irrespective of the threat from local top predators.

Our results indicate that other stimulants such as visual perception might be relevant in the sensory hierarchy of this fish and play the role of a starter in combination with chemical cues, as previously confirmed [143,159]. Interestingly, some studies conducted on other gobiids, e.g., two-spotted gobies exposed to both visual and chemical stimuli from their natural predators, have proven that in some of the cases, additional visual predator exposures can sometimes strengthen anti-predator responses to chemical cues [143]. This confirms the importance of the interaction between both types of stimulants, as has been reported in other studies [63,84,85]. Thus, we believe that further studies involving visual cues and different concentrations of chemical stimuli and application methods should be prioritised to obtain a better overview of round goby feeding behaviour and prey–predator dynamics within local ecosystems.

Since fish, including round gobies, use olfaction not only to avoid predators but also to find food, orientate themselves, and reproduce [160,161,162], we anticipated that our tested fish would actively employ olfaction for predator recognition. The failure of our hypothesis underscores the complexity of predator–prey interactions and adaptation mechanisms. While many aquatic animals recognise predators via chemical cues and respond adaptively [163], in invasive or introduced species this ability seems to be reduced, which can provide an advantage when exploiting local resources. This can potentially also lead to competitive disadvantages in predator-rich environments, but if the visual cues permit a rapid response to a threat, the cost may not be so great. Therefore, understanding the perception and management of predation threats by invasive species deepens our insight into the factors driving the rapid invasion of unfamiliar habitats by some species, while others do not exhibit such behaviours.

## 5. Conclusions

In conclusion, our study sheds light on the behaviour of the invasive round goby concerning its response to chemical stimuli from predators and injured conspecifics. Contrary to expectations, round gobies did not exhibit discernible reactions to these cues, maintaining consistent feeding behaviour regardless of predator presence. This suggests species-specific thresholds in perceiving chemical cues, highlighting the complexity of predator–prey interactions and adaptation mechanisms. Further research involving visual cues and different concentrations of chemical stimuli should be prioritized to understand better round goby feeding efficiency and behaviour while including prey–predator dynamics in invaded ecosystems. Understanding the perception and management of predation threats by invasive species is crucial for elucidating factors driving their rapid invasion of new habitats, thus contributing to more effective conservation and management strategies.

## Figures and Tables

**Table 1:** Relationships between relative gut content (*GI*) and the explanatory variables, i.e., ‘treatment’ and ‘time’. A generalised linear model (GLM) with Gaussian distribution (with the log link function applied) was used to fit and test the relationships. The significances of all relationships were tested with partial *F*-tests. Asterisks following *p*-values denote significance: * 0.05, *** 0.001.

Model	Predictor	*df*	Deviance	*F* Value	*p*-Value	
Model 1: (*GI*) ~ Treatment × Time						
	Treatment	2	0.0015	3.13	0.047	*
	Time	1	0.0034	1.56	<0.001	***
	Treatment × Time	2	0.0014	0.04	0.964	
Residual *df*: = 138						
Model 2: (*GI*) ~ Treatment + Time						
	Treatment	2	0.0015	4.69	0.011	*
	Time	1	0.0061	453.91	<0.001	***
Residual *df*: = 140						

**Table 2:** Relationship between food consumption probability of food B (eaten and offered prey was used as a response, and observations were weighted by counts of offered prey) and explanatory variables, i.e., ‘treatment’ and ‘time’. The table depicts two models: one includes the interaction between explanatory variables (Treatment × Time), while the second, additive effects of predictors (Treatment + Time). A generalised linear model (GLM) with quasibinomial distribution was used to fit and test the relationships. The significances of all relationships were tested with partial *F*-tests.

Model	Predictor	*df*	Deviance	*F* Value	*p*-Value
Food B consumption ~ Treatment × Time					
	Treatment	2	347.82	1.0178	0.3649
	Time	6	352.13	0.5607	0.7607
	Treatment × Time	12	364.15	0.5884	0.8473
Residual *df*: = 105					
Food B consumption ~ Treatment + Time					
	Treatment	2	375.73	1.8609	0.1601
	Time	6	386.55	1.1994	0.3115
Residual *df*: = 117					

## Data Availability

The data presented in this study are available on request from the corresponding author.
